# Mycelial Mattress from a Sporangia Formation-Delayed Mutant of *Rhizopus stolonifer* as Wound Healing-Enhancing Biomaterial

**DOI:** 10.1371/journal.pone.0134090

**Published:** 2015-08-14

**Authors:** Mei-Yin Chien, Ling-Chun Chen, Ying-Chen Chen, Ming-Thau Sheu, Ya-Chi Tsai, Hsiu-O Ho, Ching-Hua Su, Der-Zen Liu

**Affiliations:** 1 School of Dentistry, College of Oral Medicine, Taipei Medical University, Taipei, Taiwan; 2 School of Pharmacy, College of Pharmacy, Taipei Medical University, Taipei, Taiwan; 3 Clinical Research Center and Traditional Herbal Medicine Research Center, Taipei Medical University Hospital, Taipei, Taiwan; 4 Graduate Institute of Biomedical Materials and Tissue Engineering, College of Oral Medicine, Taipei Medical University, Taipei, Taiwan; 5 Department of Microbiology and Immunology, School of Medicine, College of Medicine, Taipei Medical University, Taipei, Taiwan; 6 Center for General Education, Hsuan Chuang University, Hsinchu, Taiwan; 7 Ko Da Pharmaceutical Co., Taoyuan, Taiwan; Northwestern University, UNITED STATES

## Abstract

A mycelial mattress of *Rhizopus stolonifer* obtained from a liquid static culture was utilized for wound dressing and biomedical use. Following screening of mutants induced by UV radiation, F6, exhibiting delayed sporangium formation was selected because its sporangium maturation exhibited a 5-day delay without significant loss of mycelial weight compared to the wild type. The sporangium-free mycelial mattress from the sporangiospore culture of F6 was treated with 1N sodium hydroxide NaOH at 85°C for 2 h to produce a sponge-like membrane named Rhizochitin. The trifluoroacetic acid hydrolysate of Rhizochitin contained 36% N-acetylglucosamine and 53% hexose respectively detected by the Elson-Morgen and phenol-sulfuric acid methods. Results indicated the wound area in rats covered with Rhizochitin was 40% less than that of the uncovered group. Rhizochitin decreased the expression of PDGF in the proliferation stage, increased the expression of TGF-β in the inflammation and proliferation stages, and increased the expression of VEGF in the inflammation and proliferation stages. Rhizochitin inhibited secretion of matrix metalloproteinase-9 on days 1, 7, 9, and 12 and matrix metalloproteinase-2 on days 3, 7, 9, and 12. It was concluded that Rhizochitin has beneficial properties of biocompatible, biodegradable, and wound healing.

## Introduction

Chitin and the deacetylated derivative of chitosan are principal parts of fungi’s cell walls.[1] Utilization of chitin and chitosan from fungal cell walls and not from crustacean sources has garnered attention in recent years.[2] This alternative source is advantageous because fungi usually possess a high growth rate, and they can be cultivated on inexpensive media.[3] Moreover, fungi do not contain calcium carbonate and other mineral salts. Thus, acid treatment during the manufacture of chitin is not necessary which leads to less environmental pollution and lower costs compared to shellfish waste processing.[4] Furthermore, production of polysaccharides from fungal sources can be effectively adjusted through controlling fermentation and processing conditions.[5]

Fungal chitin/chitosan has special properties that makes it useful for many applications including the biotransformation of chitinous fungi to single-cell proteins,[6] the removal and recovery of heavy metals and oils,[7–9] and antibacterial and antifungal activities.[10–12] However, chitin and chitosan derived from shellfish sources and their modifications were demonstrated to have wider applications as cosmetics, pharmaceuticals, and biomedical materials.[13] Furthermore, due to the biocompatible, non-toxic, and biodegradable properties of chitin/chitosan from shellfish sources, it has been developed into suture and wound dressing material with wound healing-enhancing effects.[14–16] In our previous work, cell wall components of a higher fungus, *Ganoderma tsugae*, residue as a byproduct of health food manufacturing, containing 40%~50% chitin was successfully developed as skin substitute named Sacchachitin.[17] Sacchachitin showed high biocompatibility and acceleration of wound healing by enhancing the migration and proliferation of fibroblasts[18] and keratinocytes.[19] Sacchachitin also inhibited the activity of matrix metalloproteinases (MMPs) in the wound area, and cases of human chronic wounds were shown to heal normally.[20] Early type-1 collagen deposition was found in the wound area after treatment with Sacchachitin.[19] However fruiting bodies of *G*. *tsugae* take as long as 3~4 months to harvest and the quantity of available waste residue was unstable in the past due to market fluctuations of health foods. Therefore, a more readily obtainable source of chitinous materials for this medical purpose is needed.

Fast-growing lower fungi, especially species of Zygomycetes, are one of the choices to produce chitin/chitosan as revealed above.[1] It was reported that chitosan in and/or chitin osan are a main major structural biopolymers in cell walls of three genera of Zygomycetes fungi including *Mucor*, *Absidia*, and *Rhizopus*.[1] Among them, *M*. *indicus*, also known alternatively recognized as *M*. *rouxii*, *Chlamydomucor rouxii*, and *Amylomyces rouxii*, is the most essential member. *Mucor indicus* is currently used to produce several homemade food and beverages particularly in Asia. Those fungi are generally regarded as safe (GRAS) and have been employed as a safe dietary source for rats and fish.[2] *Mucor rouxii* is also the most commonly studied fungal source of chitin and chitosan, which was proven to be safe for several biotechnological applications.[2] Moreover, these fungi form a porous spongy mycelial mattress on the surface of liquid medium that can be easily separated from the culture medium to make a natural nonwoven material for wound dressings without further fabrication. Thus, the manufacturing process is considerably shorter. However, there are still some drawbacks with the use these fungi in wound-dressing production. One is that Zygomycetes usually produce dark-colored sporangia, which gives the product an unsightly appearance. Another concern is that these fungi emit a large number of sporangiospores into the air and these might cause opportunistic mycoses during cultivation.

In the present study, *Rhizopus stolonifer* (Ehrenberg: Fries) Vuillemin, the common black bread mold isolated from koji for rice liquor was used instead of *Mucor*. That is because some species of *Rhizopus* are believed to be safer, and they are extensively applied as a source of amylase or directly as food in the food industry. On the other hand, two strategies are employed to produce sporangia-free mycelial mattresses. One is to select an optimal culture condition for the production of mycelial mattress with the highest mycelial mass and minimal sporangia formation. The other approach is to screen a sporangium formation-delayed mutant (SFDM), in which mycelium growth reaches a plateau before sporangiospore formation. In consequence, spore emission during cultivation can be avoided. To evaluate the effect of the sponge-like mattress on wound-healing enhancement, an animal model with a full-thickness lesion was designed by measuring MMP activity and growth factor contents in the wound area. Data reported in this paper may be useful for optimizing sporangia-free material and possible use in the care of skin wounds.

## Materials and Methods

### Culture

*Rhizopus stolonifer* var. *stolonifer* (Ehrenberg; Fries)Vuillemin BCRC 32002 was originally isolated fromfermented rice grains bought from Taiwan Tobacco & Liquor Corporation byProf. Su. The culture was stored at 4°C on potato dextrose agar (PDA, BD Difco, Franklin Lakes, NJ, USA). The study did not involve endangered or protected species.

### Tests of optimal culture conditions

Sporangiospore suspensions with a spore concentration of 10^7^/ml were prepared by adding a sterilized 0.1% Tween 80 solution to agar slants followed by sonication for 3 min. Spore suspensions (1.5 x 10^7^/flask) were inoculated into 250-ml flasks containing 100 ml potato dextrose broth (PDB, BD Difco), and PDB with surplus glucose of 1.0%, 1.5%, 1.75%, 2.0%, 2.25%, 2.5%, 2.75%, 3.0%, and 4.0%. In the other group, PDB was added with a surplus peptone of 1.0%, 1.25%, 1.5%, 1.75%, 2.0%, 2.25%, 2.5%, 2.75%, 3.0%, and 4.0%. Each treatment of medium was conducted in triplicate.

To determine an optimal growth temperature, PDB with 2.0% surplus glucose was prepared and cultured in the same manner except the incubation temperature was set to 24, 29, and 37°C. To determine the inoculation density, three spore concentrations were employed of 10^7^, 5 x 10^7^ and 10^8^/flask, and the medium consisted of PDB with 2.0% surplus glucose.

All cultures were incubated statically at 24°C for 4, 8, 12, and 16 days after inoculation. After a desirable growth time, the mycelial mattress was separated by pouring out the broth and washing with distilled water until the wash water was clear and lyophilized. The dried mattress was weighed, and the number of sporangia was counted under a dissection microscope (Nikon, Tokyo, Japan). The average of three randomly selected fields of the mattress was counted at a fixed magnification (60x).

### Deproteinization

To 1 g of mycelial mattress 300 ml of 1 N KOH was added, and the mixture was heated to 90°C, then kept for 3 h. The mattress was washed with distilled water until the wash water was a neutral pH 7.0. The deproteinized mattress was lyophilized and weighed.

### Mutagenesis

Sporangiospore suspensions of 10^6^/ml in 12-cm-diameter Petri dishes were irradiated under UV light (15 W) at a distance of 44 cm for 200 s to reach a 95% lethal rate. The dishes were kept in the dark for 1 h. Surviving spores were diluted and grown on PDA plates for germination, and colonies without sporangium were selected at day 8 after irradiation. On day 16 after irradiation, the sporangia of the mutants had reached maturation, and spores were collected to cultivate the mycelial mattress.

### Hydrolysis

Samples (5 mg) of a deproteinized mycelial mattress from wild type (WT) and mutants, and different culture conditions were hydrolyzed in 3 ml of trifluoroacetic acid (Riedel-deHaen, Seelze, Germany) in sealed ampoules at 110°C for 16 h. Samples were then evaporated to dryness in a desiccator using NaOH pellets and silica gel as the adsorbent under reduced pressure. The dried hydrolysates were dissolved in 5 ml of distilled water for further analysis.

#### Detection of N-acetyl-glucosamine

A portion of the described hydrolysates solution was analyzed by thin-layer chromatography (TLC). The solvent system for the TLC analysis was ethyl acetate: isopropanol: water (1: 1: 3), and visualizing agents were naphtharesorcinol for hexoses and Elson-Morgan reagent for N-acetyl glucosamine. Another portion (2.5 ml) was used to determine the N-acetyl-glucosamine concentration of hydrolysates. The Elson-Morgan procedure as modified by Chaplin [21] was used. Chitin (Sigma-Aldrich, St. Louis, MO, USA), glucosamine, and N-acetyl-glucosamine were employed as controls. The calibration curve was prepared from 0.1 to 1.0 mg of N-acetyl-glucosamine.

### Wound-healing studies

This animal experiment was approved by the Institutional Animal Care and Use Committee of Taipei Medical University (approval no.: LAC-100-0101), and was in compliance with the Animal Welfare Act. Wistar rats were 3-month old and weighed 300±30 g, and were obtained from the National Animal Center, Taipei, Taiwan. Before the study, rats were sedated with Zoletil 50 (0.5 ml/rat) dispensed in phosphate-buffered saline (PBS), and the dorsal hair was shaved with an electric razor. Three identical areas (6 mm in diameter) on each side 10 mm off the central line of dorsal were marked and full-thickness wounds of six were removed. After the blood residue was cleaned off, the wounds were randomly enclosed with the dressing materials prepared. BESCHITIN-W (Unitika, Kyoto, Japan), Sacchachitin, and cotton gauge were included for comparisons. During the study period, the rats stayed in the room with an air-conditioned and a 12/12-h dark/light cycle. Regular rat foodstuff and tap water were supplied. Weighing and observation were performed once a week to check the health of the rats. Zoletil was used to alleviate pain suffering when needed. The experiment was repeated in three animal numbers. In the end, rats were humanely sacrificed by carbon dioxide inhalation euthanasia in the laboratory of Animal Center, Taipei Medical University.

#### Preparation of histological specimens

Skin samples of full-thickness were taken from the original wound of the rats with a punch of 8 mm in diameter. Prior to sampling, rats were anesthetized with Zoletil 50 on days 1, 2, 4, 8, and 14 after skin injury. Specimens were cut into two equal portions and frozen at -80°C. The first portion was fixed for 24 h in PBS containing 10% formaldehyde and then implanted with paraffin after the dehydration process by treatment with an alcohol gradient and xylene. Samples were cut on a microtone into 6-mm-thick serial sections, dewaxed, and stained with hematoxylin and eosin (H&E) for visualization. Changes in the wound area were recorded and analyzed by a software PIA (Power Image Analysis) system attached to a Finescope Series Video Microscope (Model FS-180), designed by Ching-Hsing Computer-Tech (Taipei, Taiwan).

#### Preparation of rat skin biopsy extraction

Tissue samples were sliced with razor and homogenized in 500 ml of extraction buffer (10 mM Tris at pH 7.4, 150 mM NaCl, and 1% Triton-X 100) at 4°C employing an ultrasound homogenizer (UP-50H, Hielscher Ultrasonics GmbH, Teltow, Germany). Homogenates transferred to 1.5-ml Eppendorf tubes were centrifuged at 13,000×*g* for 10 min, and the supernatant was saved at -80°C until being examined. Aliquots from each biopsy were individually subjected to analyzed with enzyme-linked immunosorbent assays (ELISAs) for transforming growth factor (TGF)-β1, platelet derived growth factor (PDGF), and vascular endothelial growth factor (VEGF), and gelatin zymography.

#### Growth factors

Total protein contents and levels of TGF-β1, PDGF, and VEGF in the biopsy extracts were determined by ELISAs, following the manufacturers’ protocol for proteins (Pierce Biotechnology, Rockford, IL, USA) and growth factors (Quantikine, R&D Systems, Minneapolis, MN, USA).

#### Gelatin zymography

Aliquot samples were examined by gelatin zymography following the method described by Snoek-van Beurden and Von den Hoff.[22] Biopsy sample extracts (10 ml) were mixed with 10 ml of 2x sample buffer (0.125 M Tris buffer at pH 6.8, 20% (v/v) glycerin, 4% (w/v) SDS, and 0.005% bromophenyl blue) for ten mins at room temperature. The standard was MMP-9 (Sigma-Aldrich).

#### Statistical analysis

The mean ± standard deviation (SD) was reported for all results. Statistical significance was determined by a one-way analysis of variance (ANOVA)(PASW Statistics 18.0). A statistically significant value of *P* < 0.05 was set.

## Results

### Optimization of culture conditions for *R*. *stolonifer*

**Fig 1:**
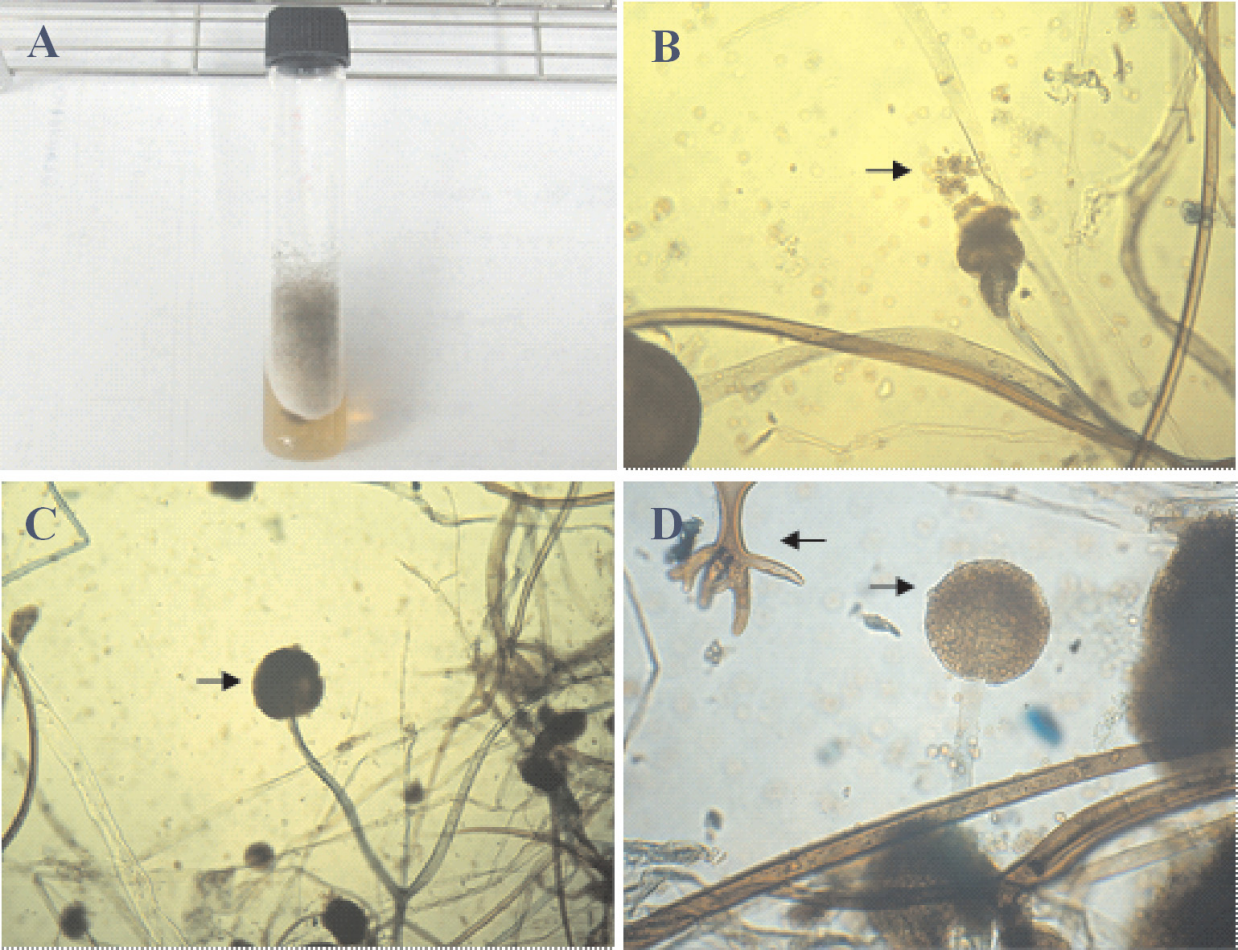
*Rhizopus stolonifer*. (**a**) Cultured at 29°C on the surface of potato dextrose agar (PDA) for 7 days; (**b**) sporangiophore (100x); (**c**) sporangia (100x); (**d**) sporangia (200x).

**Fig 2:**
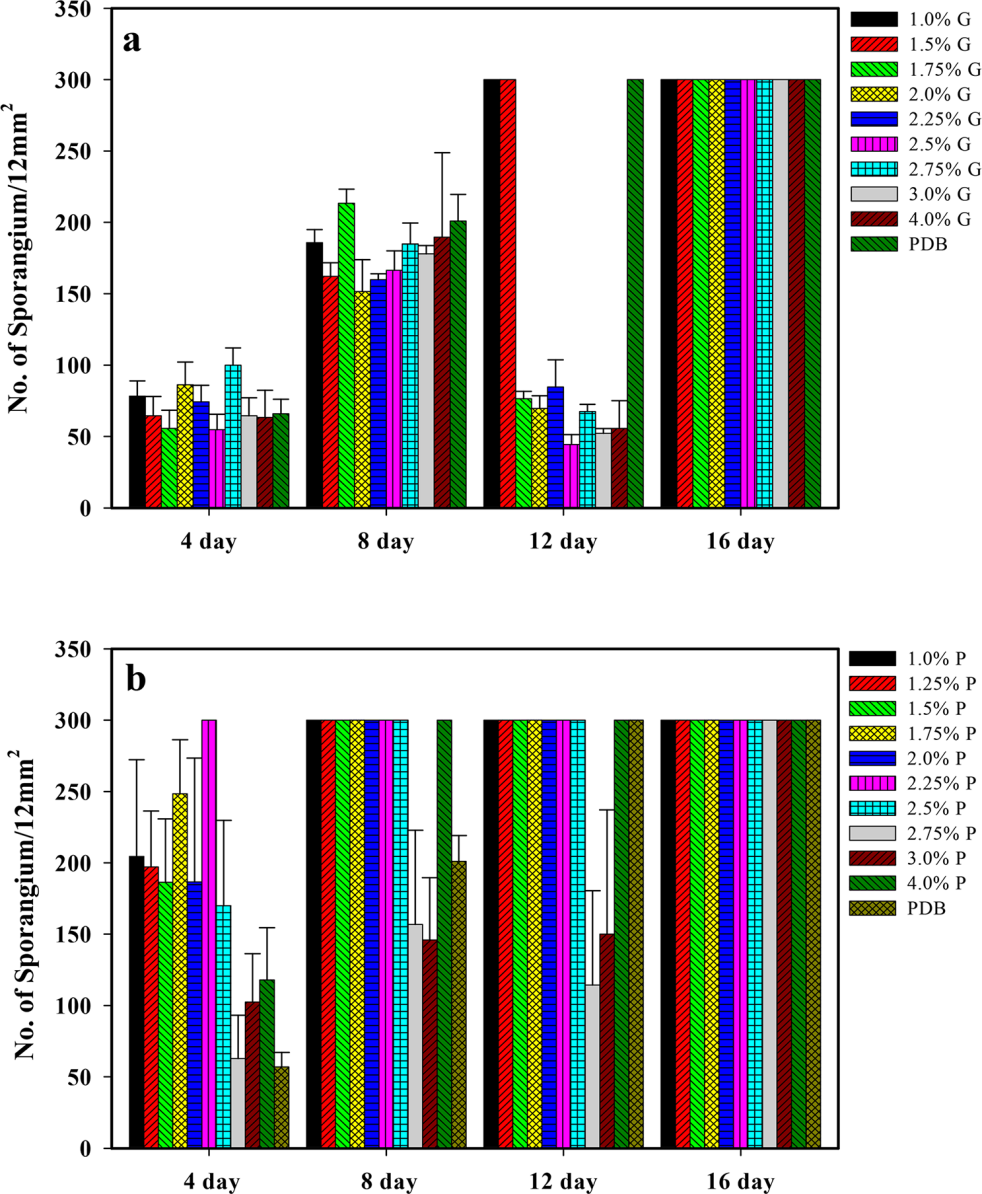
Sporangium with respect to the time in the spore suspension (1.5x10^7^/flask) inoculated in 250-ml flasks containing 100 ml potato dextrose broth (PDB), with 1%~4% surplus glucose (a) and 1%~4% surplus peptone (b). G: surplus glucose; P: surplus peptone

**Fig 3:**
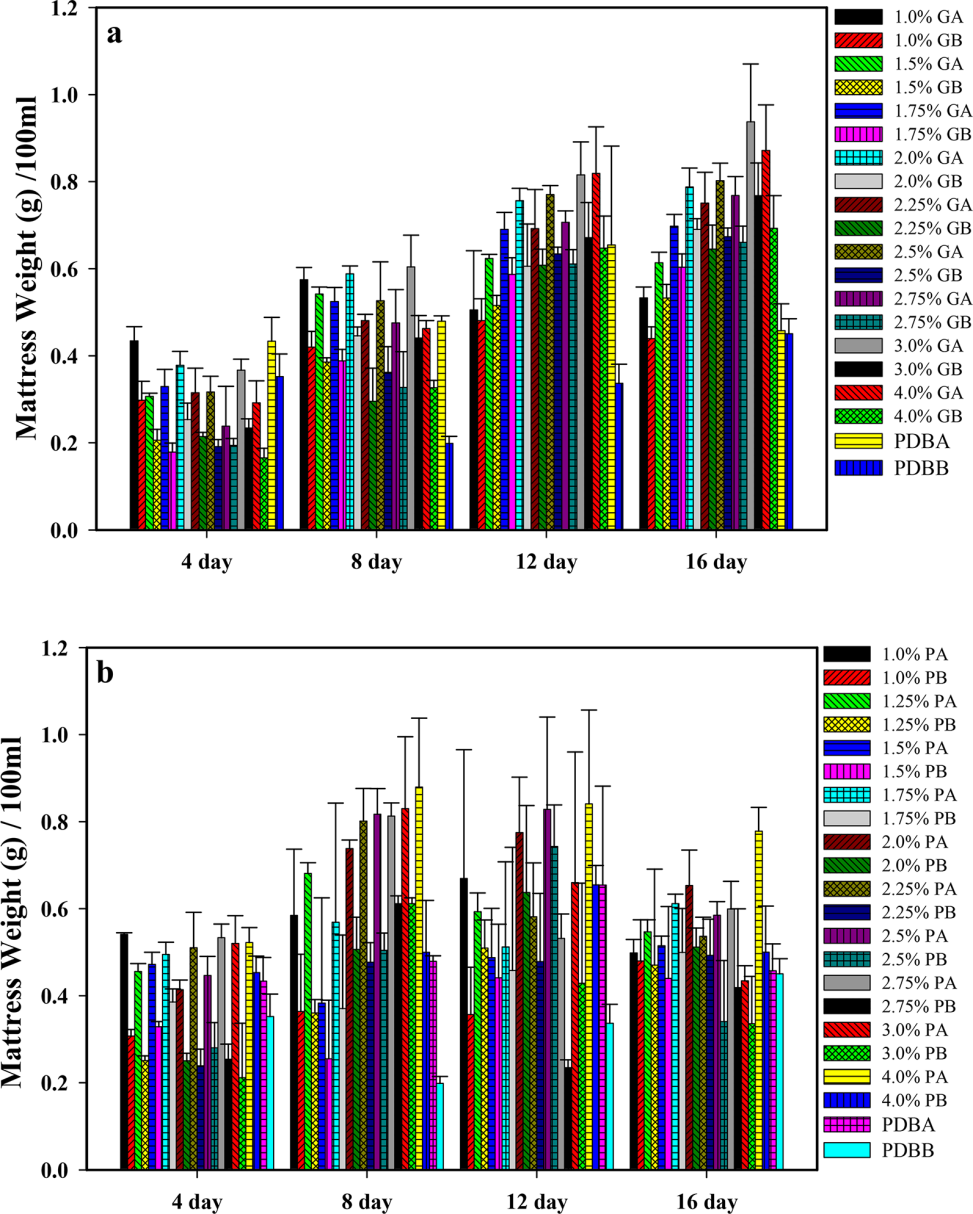
(a)1%~4% surplus glucose and (b)1%~4% surplus peptone. Dried mattress weight with glucose (GA) or peptose (PA); deproteinized mattress weight with glucose (GB) or peptose (PB) of mycelia mattresses formed with respect to the time cultured in potato dextrose broth with glucose (PDBA) or peptose (PDBB).

**Fig 4:**
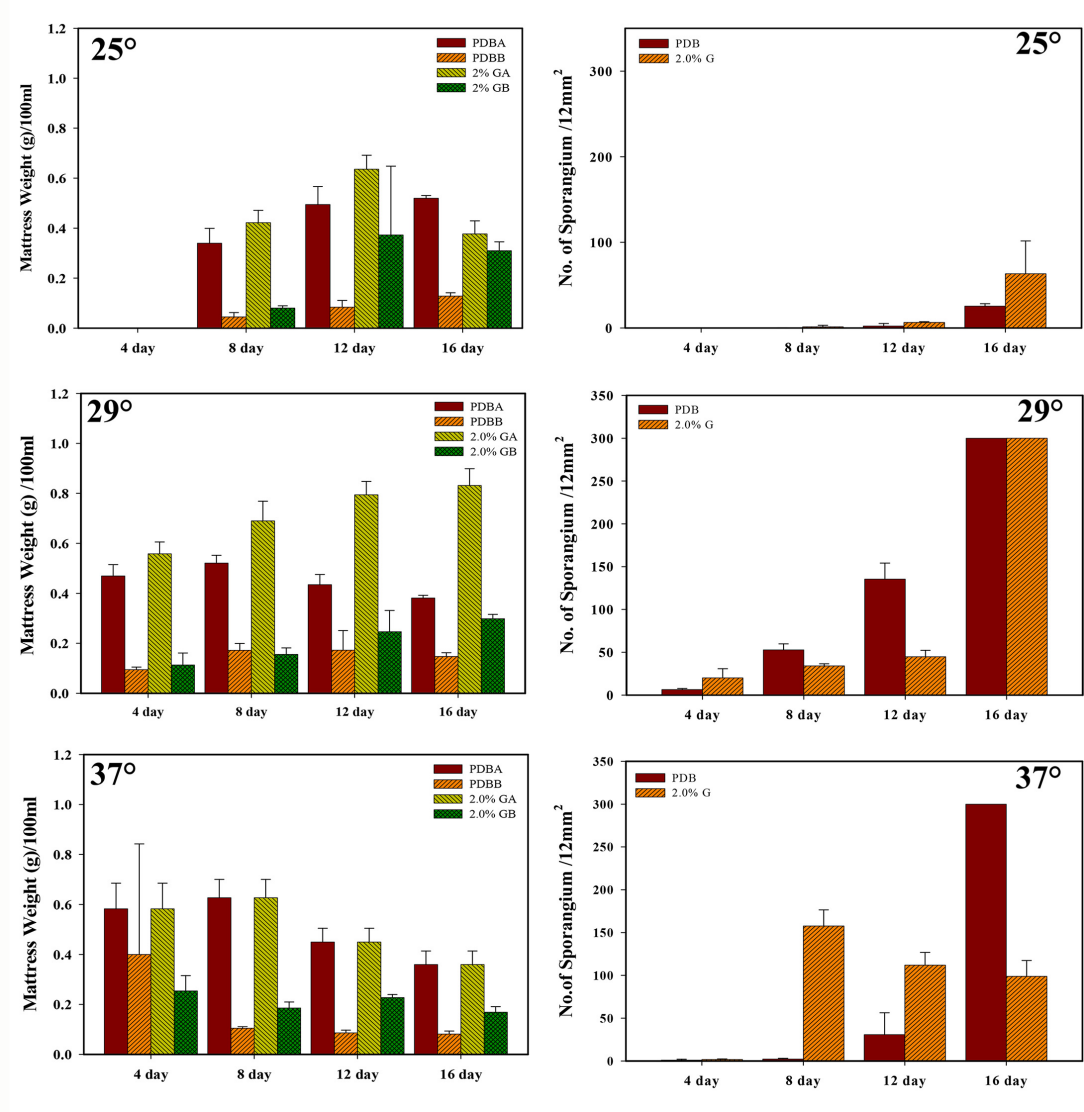
Dried mattress weight (right column) and number of sporangium (left column) of mycelia mattresses formed with respect to the time cultured in potato dextrose broth (PDB), with 2% surplus glucose at three different temperatures of 25 (a,b), 29 (c,d), 37°C (e,f).

**Fig 1A** shows the culture of *R*. *stolonifer* at 29°C on the surface of PDA for 7 days. Sporangiophore after the 7-day culture is illustrated in **Fig 1B**. Sporangia can clearly be observed at a magnification of either 100× (**Fig 1C**) or 200× (**Fig 1D**). In order to determine suitable culture conditions for *R*. *stolonifer* to obtain mycelia mattresses formed with the highest amount of dried and deproteinized mattress weights, the sporangium in the spore suspension (1.5x10^7^/flask) inoculated into 250-ml flasks and No. sporangium/12 mm^2^ (**Fig 2**) and dried and deproteinized mattress weights for mycelia mattress formation (**Fig 3**) were first compared by culturing *R*. *stolonifer* in varying combinations of glucose (PDB surplus 1%~4%, **Figs 2A** and **3A**) or peptone (PDB surplus 1%~4%, **Figs 2B** and **3B**). **Fig 4** further compared No. sporangium/12 mm^2^ (**Fig 4A, 4C, and 4E**) and mattress weights/100ml (**Fig 4B, 4D, and 4F**) for mycelia mattress formation at three different temperature (25°C: **Fig 4A and 4B**, 29°C: **Fig 4C and 4D**, and 37°C: **Fig 4E and 4F**) for different period of time (4, 8, 12, and 16 days). After comparison, the best conditions for culturing *R*. *stolonifer* giving the smallest amount of spore generation and the highest weight of the dried mattress and deproteinized mattress were culturing *R*. *stolonifer* in PDB medium with a 2% surplus of glucose at 29°C for 8 days.

To further screen for SFDMs, in which mycelium growth reaches a plateau before sporangiospore formation to avoid spore emission during cultivation, sporangiospore suspensions were irradiated with UV light (15W) at a distance of 44 cm for 200 s to reach a 95% lethal rate. The dish was kept in the dark for 1 h. Surviving spores were diluted and grown on PDA plates for germination, and colonies without sporangium were selected at day 8 after irradiation. On day 16 after irradiation, the sporangia of the mutants had reached maturation, and spores were collected to cultivate mycelial mattresses. Sixteen mutants were selected for comparison.

**Fig 5:**
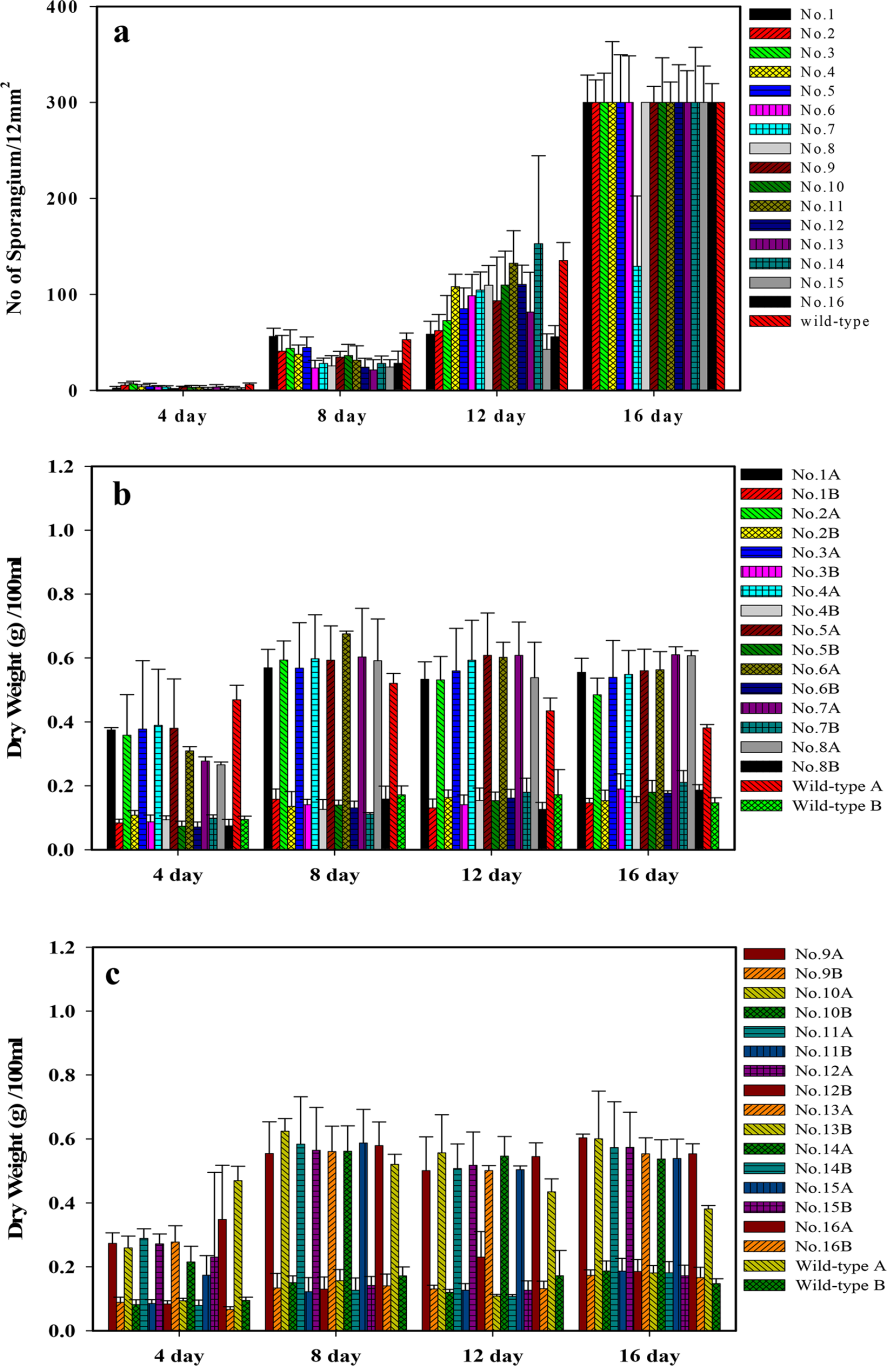
Number of sporangium (a) and dried mattress weight (b: Dried mattress weight; c: deproteinized dried mattress weight) for the wild type and its 16 mutants induced by UV radiation cultured in potato dextrose broth (PDB) with 2% surplus glucose at an incubation temperatures of 29°C for four different time periods.

**Fig 6:**
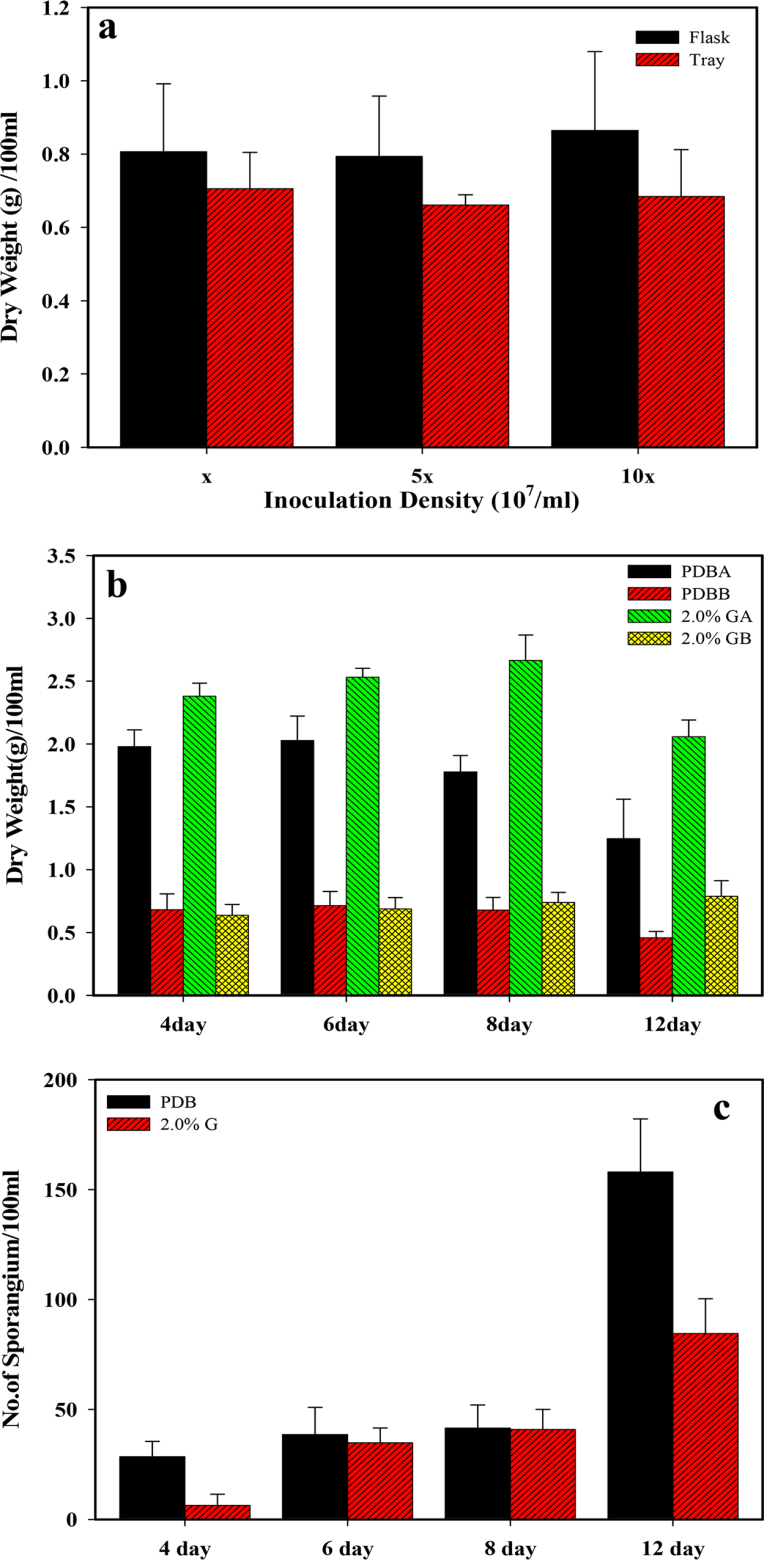
Dry mattress weight for mutant no. 6 cultured in a flask or tray with three different inoculation densities (a), and non-deproteinized (A) and deproteinized (B) dried mattress weight (b) for mutant no. 6 cultured in potato dextrose broth (PDB) or PDB with 2% surplus glucose (G) for different time periods. Number of sporangium (c) of mutant no. 6 cultured in PDB or PDB with 2% surplus glucose for different time periods.

**Fig 7:**
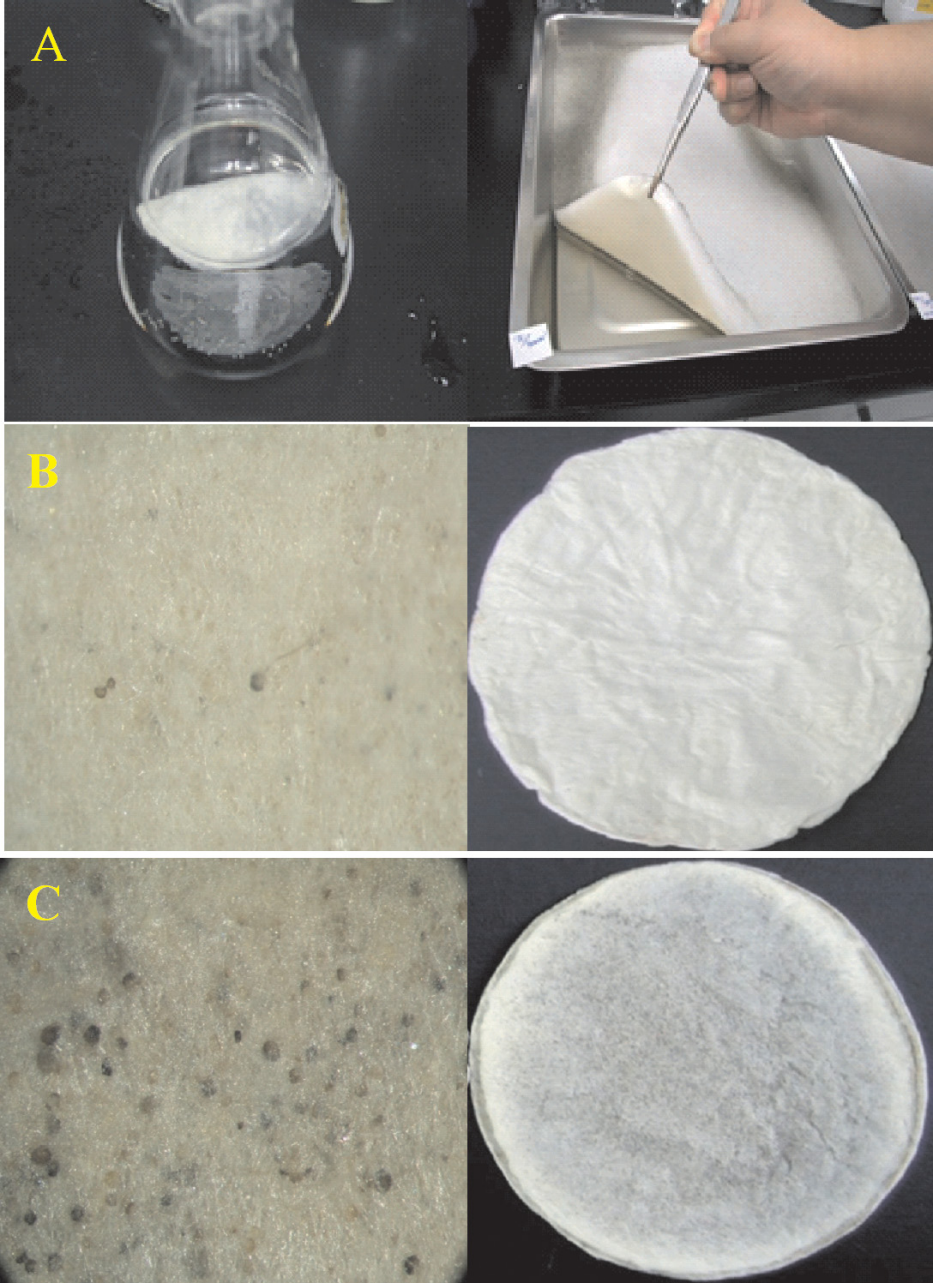
Mutant no. 6 cultured in a flask or tray (a); the appearance of the dried mattress (right column) and deproteinized dried mattress (left column) for mutant no. 6 (b) and wild type (c) cultured in potato dextrose agar (PDA) with 2% surplus glucose at 29°C for 8 days.

**Fig 5** shows the comparison of the number of sporangium (Fig 5A), dried mattress weight (Fig 5B), and deproteinized dried mattress weight (Fig 5C) between the WT and its 16 mutants induced by UV radiation by culturing them in PDB with 2% surplus glucose at an incubation temperature of 29°C for four different time periods. Based on the same criteria, the new strain labeled as *R*. *stolonifer* F6 was found to be the most optimal. **Fig 6A** further demonstrates that F6 could be cultured in flasks and on trays with no effect of the inoculation density on the dried mattress weight formed. The dried mattress weight (either non-deproteinized or deproteinized) formed by culturing F6 on PDB with 2% surplus glucose at 29°C was higher than that when culturing on PDB only (**Fig 6B**). But in both culture media, F6 demonstrated a delay of 12 day with fewer spores occurring as shown by **Fig 6C,** and the dried mattress weight reached the highest amount. We concluded that delay in spore formation and lower occurrence of spores were beneficial properties of F6, and a new biomaterial in a sheet form (designated Rhizochitin) was produced as shown in **Fig 7A**, on which fewer black spots appeared (**Fig 7B**) and the dried mattress (**Fig 7C**) was denser than that produced by the WT.

### Chemical properties of Rhizochitin

**Fig 8:**
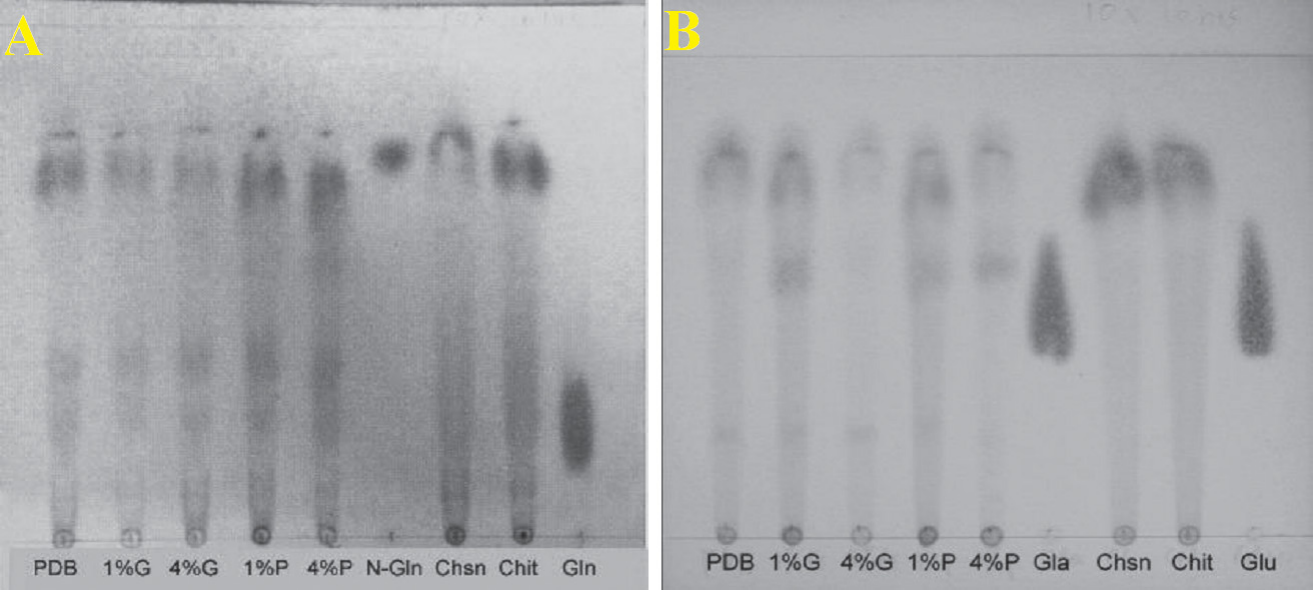
a: Thin layer chromatography (visualized with Elson-Morgan reagent) after 16 hr digestion of trifluoroacetic acid for Mutant F6 cultured in PDB or surplus (from left to right) 1.0 or 4% glucose, 1.0 or 4% peptone (lane 1–5, lane 6: N-acetyl glucosamine, lane 7: chitin, lane 8: chitosan, lane 9: glucosamine); b: Thin layer chromatography (visualized with Naphthoresorcinol/ethanol/sulphuric acid reagent) after 16 hr digestion of trifluoroacetic acid for Mutant No. 6 cultured in PDB or surplus (from left to right) 1.0 or 4% glucose, 1.0 or 4% peptone (lane 1–5, lane 6: galactose, lane 7: chitin, lane 8: chitosan, lane 9: glucose).

Rhizochitin is the mycelia mattress of *R*. *stolonifer* F6. Chemical properties of Rhizochitin (5 mg) were examined by hydrolysis with 3 ml trifluoroacetic acid. After 16 h of hydrolysis, contents of N-acetylglucosamine and hexose of Rhizochitin were determined. By TLC as shown in **Fig 8**, Rhizochitin showed the presence of N-acetyl glucosamine, a unit of chitin, when cultured on PDB with 1% and 4% surplus glucose and 1% and 4% peptone.

To ensure the quantity of N-acetyl glucosamine in Rhizochitin, it was examined by an Elson-Morgan assay as shown in **Fig 8A**. By detecting light absorption at 585 nm with comparison to a standard curve (not shown), it was determined that Rhizochitin contained approximate 36% N-acetyl glucosamine. On the other hand, the content of hexose in Rhizochitin was confirmed to be 53% using the phenol-sulfuric acid method as shown in **Fig 8B**. It was concluded that 36% of total polysaccharides in Rhizochitin was N-acetylglucosamine/glucosamine, with 53% hexose and the rest unknown saccharides.

### Animal wound-healing study

**Fig 9:**
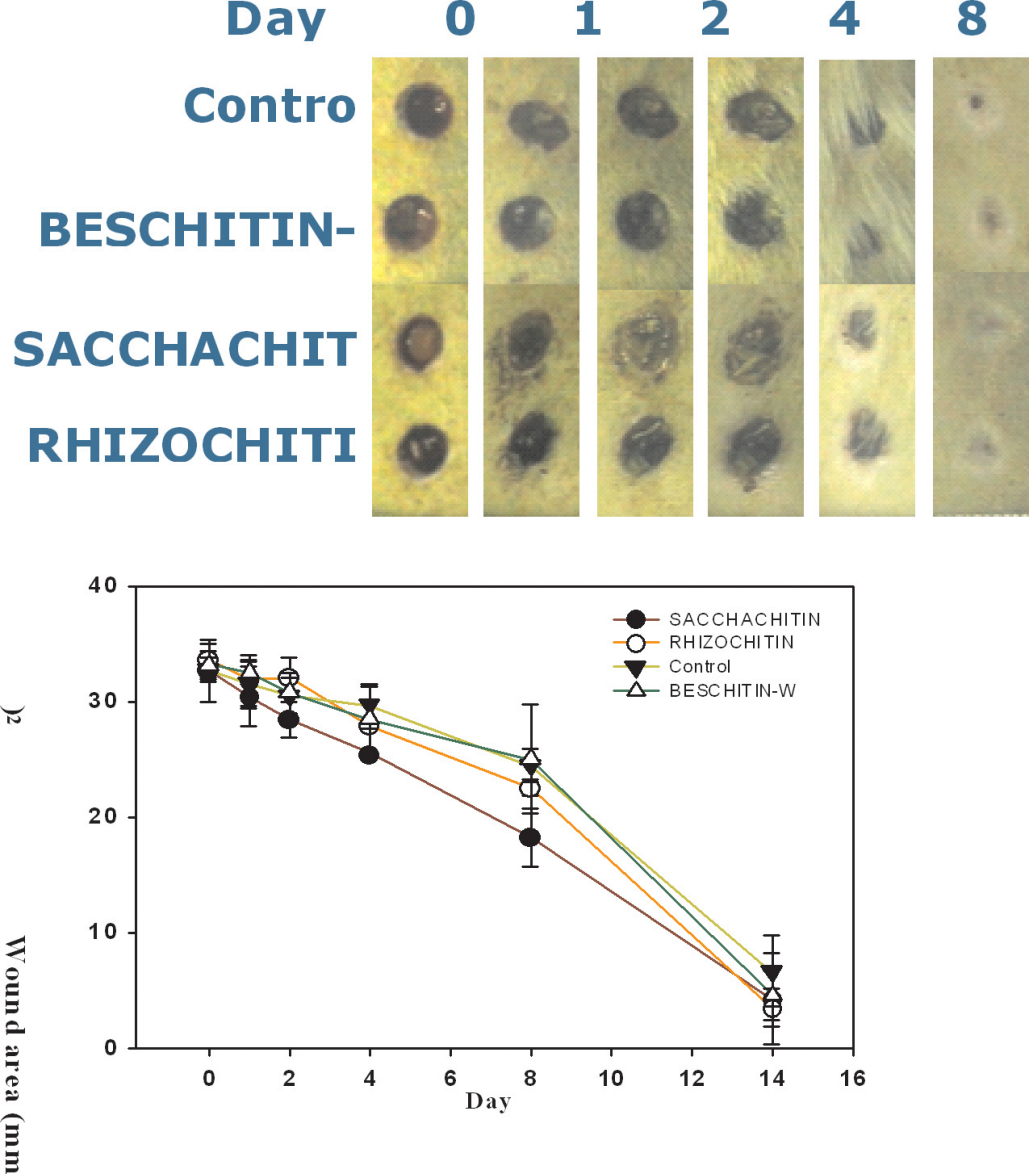
Plots of changes in the wound area versus time for those covered with cotton gauze as the control.

**Fig 9** shows the area changes in the wound enclosed with Rhizochitin, Sacchachitin, BESCHITIN-W, and the control (cotton gauze). Rhizochitin had an obvious healing-promoting effect on the wound on days 4, 8, and 14, and its healing speed was faster than that of the control. Sacchachitin was better than the control on days 1 to 14, whereas the effect of BESCHITIN-W did not greatly differ from that of the control. In other words, the healing effect of Rhizochitin was better than that of BESCHTIN-W and comparable to that of Sacchachitin. Overall, Rhizochitin could be utilized as an equivalent to Sacchachitin as a good biomaterial for wound healing.

### Growth factors in the wound

**Fig 10:**
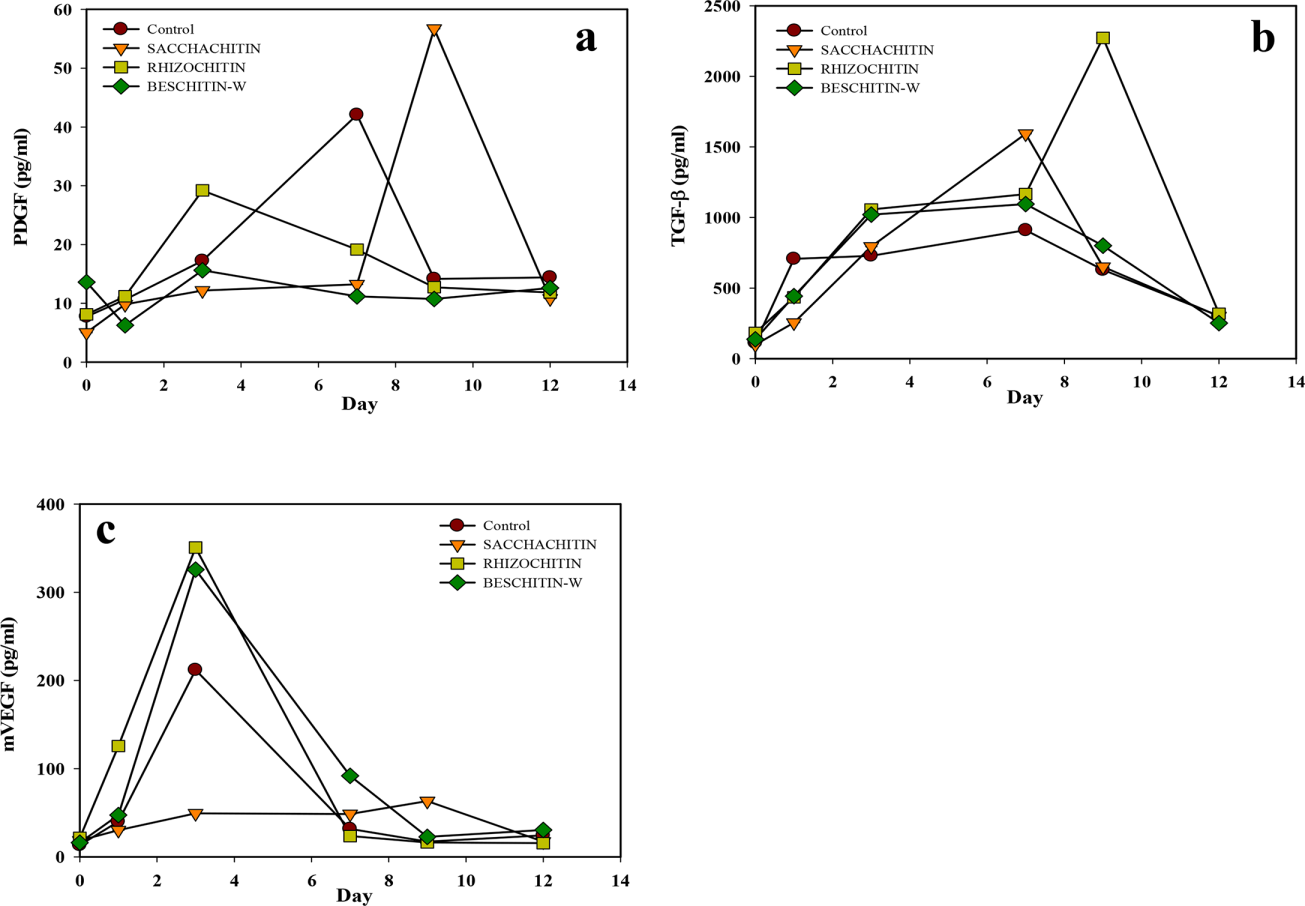
Changes in the amounts of growth factor (PDGF) (a), transforming growth factor (TGF)-β (b), and vascular endothelial growth factor (VEGF) (c) versus time in the wound area covered with cotton gauze, Sacchachitin, Rhizochitin, or BESCHITIN-W.

The wound-healing process was conveniently divided into three stages that overlap in a continuous and temporal way: inflammatory stage (beginning to 2~5 days), proliferative stage (2 days to 3 weeks), and remodeling stage (3 weeks to 2 years). A variety of different growth factors (PDGF, TGF-β, VEGF, etc.) control each of stages in the healing procedure.[23] The time course of growth factor appearance in the histological sample from the wound area was assayed by ELISAs, and results are illustrated **in Fig 10**. For both Rhizochitin and BESCHITIN-W, the expression of PDGF (**Fig 10A**) decreased in the proliferation stage, but contrarily increased with Sacchachitin. **Fig 10B** shows the increasing expression of TGF-β in the inflammation and proliferation stages, but it was inhibited by Sacchachitin in the inflammation stage. VEGF increased in the inflammation and proliferation stages with all biomaterial treatments as shown by **Fig 10C**. It could be concluded that Sacchachitin enhanced the secretion of growth factors and promoted healing wound in the late stage, and Rhizochitin inhibited inflammation in the early stage.

**Fig 11:**
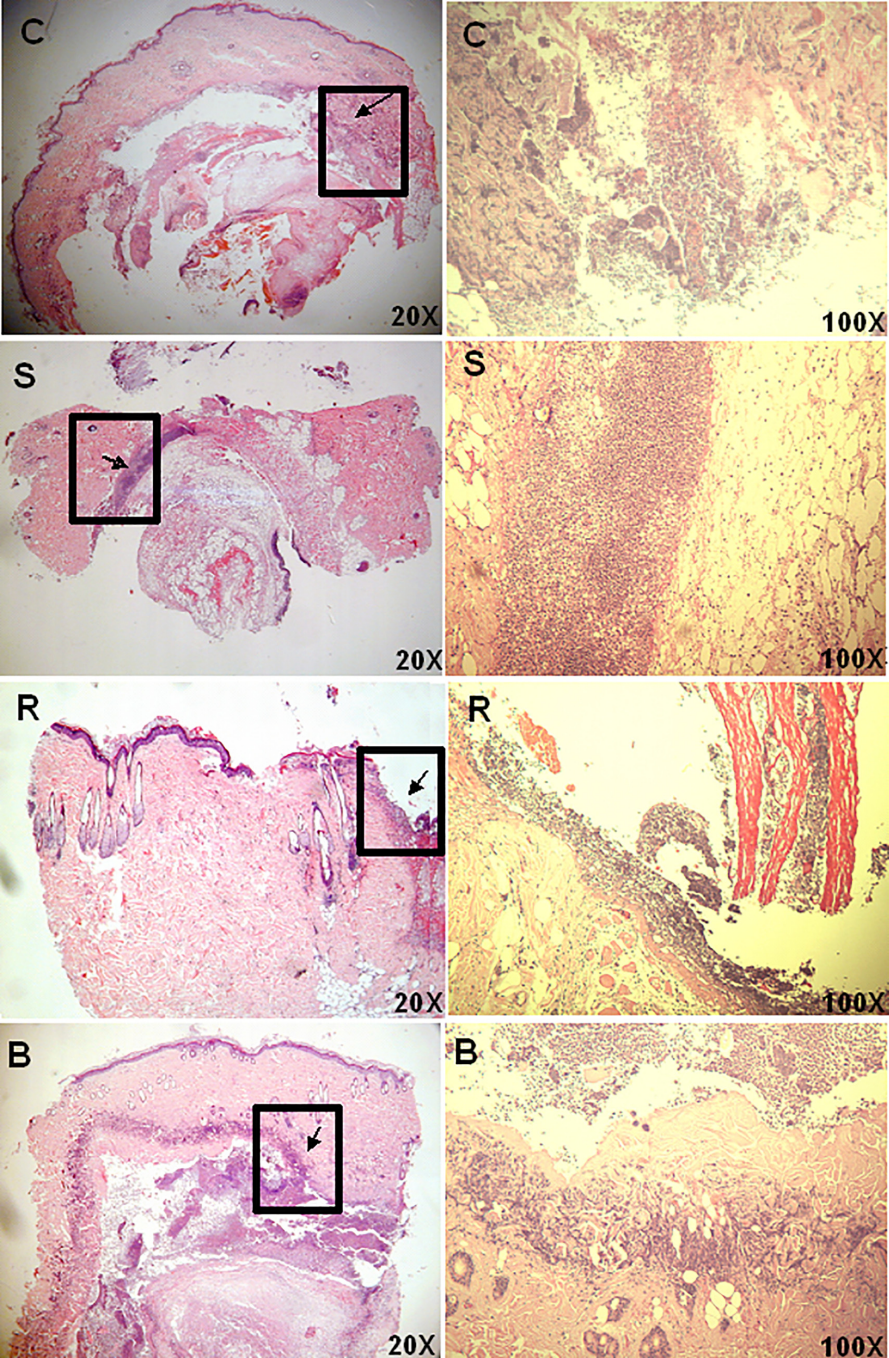
Histological staining with hematoxylin and eosin (H&E) for the wound area covered for 3 days with the control (C), cotton gauze, Sacchachitin (S), Rhizochitin (R), or BESCHITIN (B).

Histological staining of samples recovered on day 3 (during the inflammation stage) is shown in **Fig 11,** and it demonstrates that neutrophils inducing inflammation had accumulated between the epidermis and dermis (as indicated by an arrow) for those wounds covered with the control, Sacchachitin, and BESCHITIN-W, whereas they had only accumulated on the surface of the epidermis for wounds covered with Rhizochitin. This indicates that Rhizochitin inhibited the overexpression of inflammatory reactions. Overall, it was able to conclude that Sacchachitin could stimulate the secretion of growth factors responsible for proliferation and healing to promote wound healing in the late stage, while Rhizochitin inhibited the excess extent of inflammatory reaction in the early stage.

### Expressions of MMP-2 and MMP-9 in the wound

**Fig 12:**
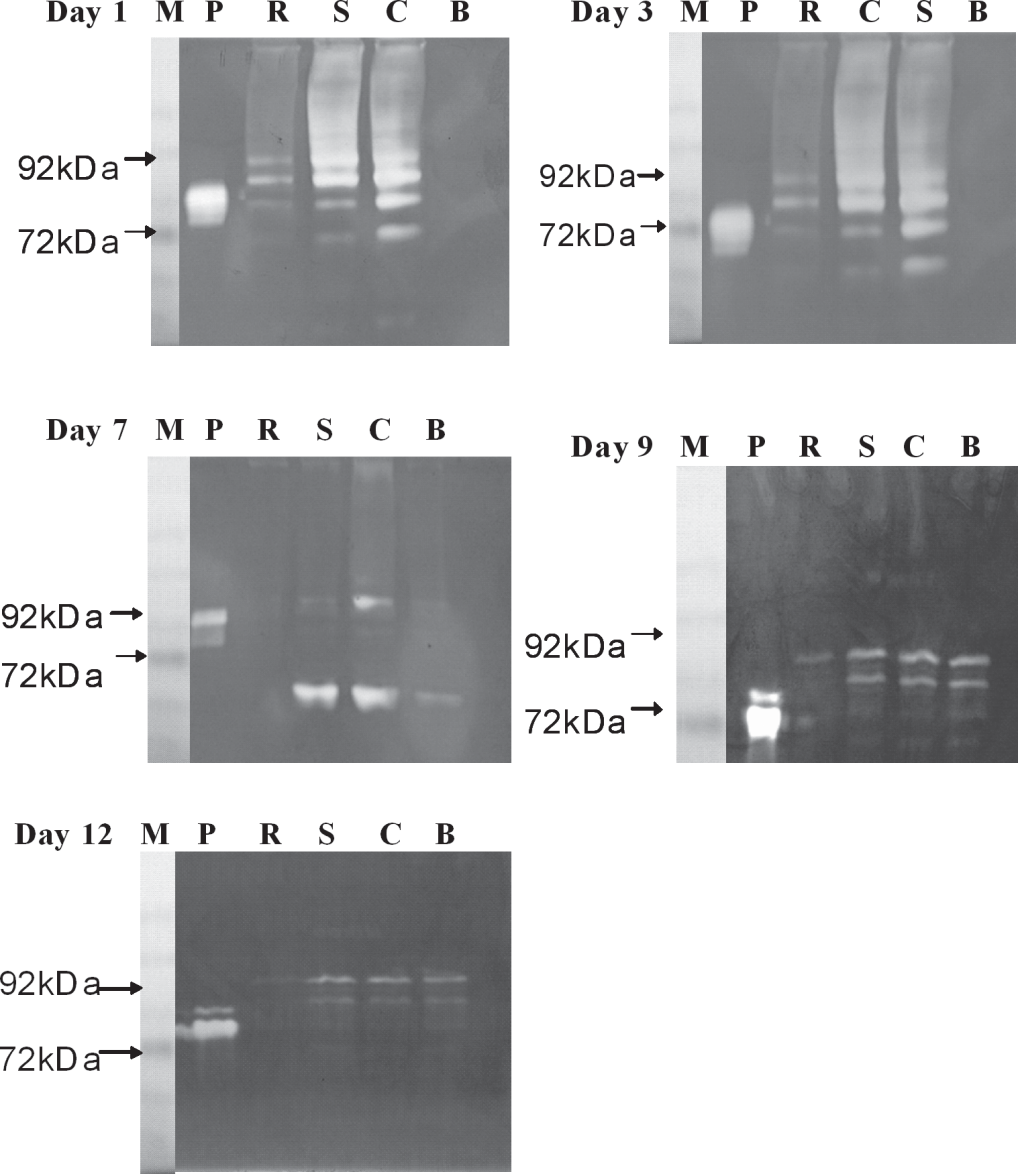
Detection of matrix metalloproteinase (MMP)-9 (92 KDa) and -2 (72 KDa) by gelatin zymography in extracts from the wound area covered for different time periods with the control, cotton gauze (C), Rhizochitin (R), Sacchachitin (S), BESCHITIN-W (B). (**M**, marker; **P**, Protease standard).

Metalloproteinases are recognized as essential participants in regulatory mechanisms in all stages of wound healing. During inflammation, MMPs can activate mediators (cytokines and chemokines) by detaching them from the cell surface (epithelium, endothelium, fibroblasts, etc.) or by enhancing their activity, or degrade them, thus inhibiting inflammatory signals. Furthermore, MMPs can cleave cell-cell junction components and cell-matrix contacts within the epithelium to stimulate reepithelialization. Furthermore, MMPs are directly involved in the restoration of scar ECM by proteolytic degradation of collagens.[24] Therefore, MMP-9 and -2, a kind of gelatinase responsible for reducing and remodeling the ECM, were examined during the wound-healing process. Zymographic analysis of MMP-9 and -2 as shown in **Fig 12** revealed that both appeared on days 1, 3, 7, and 9 in the control, indicating that both activities were inhibited during the inflammation and proliferation stages of the wound-healing process. It further illustrated that Rhizochitin inhibited secretion of MMP-9 at day 1, 7, 9, and 12 and MMP-2 at day 3, 7, 9, and 12. For Sacchachitin, MMP-9 was inhibited on days 7, 9, and 12 (in the inflammation and remodeling stages) and MMP-2 only appeared on day 7. BESCHITIN inhibited the expression of MMP-9 only on day 1 (in the inflammation stage). It could be concluded that all biomaterials in this study promoted wound healing by decreasing the overexpression of MMPs.

## Discussion

The ideal wound dressing should have the following characteristics: (1) comfortable in place over the wound, (2) impermeable to bacteria, (3) able to maintain a high moistness at the wound site while eliminating excess exudates, (4) able to protect the wound from further trauma, and (5) cost effective and easily produced. Rhizochitin comes from mycelial mattress of *R*. *stolonifer* F6, is a new wound dressing that can be rapidly produced, and does not need to be woven or bleached. This new biomaterial is a sponge-like porous material with a strong ability to absorb water. After absorbing water, it becomes very soft but does not lose its tenacity. Because of this, Rhizochitin can tightly adhere to wound sites. In addition, it can maintain a humid environment at the wound site to foster a better repair environment. On the other hand, Rhizochitin is a chitin-like biomaterial with some beneficial properties, e.g., biocompatible, biodegradable, and nontoxic, and it accelerates wound healing.

In animal experiments of this study, the healing rate with Rhizochitin was 35% better than the control, equivalent to BESCHITIN, but worse than Sacchachitin. This is because chitin is not the only component of Rhizochitin, but it also has some other polysaccharide components. Examination of changes in the amount of PDGF, TGF-β, and VEGF during different healing stages demonstrated that Rhizochitin could inhibit inflammation and help wound healing in the late stage. As reported by Werner et al. [25], PDGF was the initial growth factor shown to be chemotactic for neutrophils, monocytes, and fibroblasts, thus travelling to a healing skin wound. Additionally, it enhanced the proliferation of fibroblasts and their production of the ECM. Ultimately, it stimulated fibroblasts to make collagen matrix contract and induced the myofibroblast phenotype in them. TGF-βs were demonstrated to be mutagenic for fibroblasts, however most of those cells were inhibited of proliferation. Furthermore, TGF-βs are powerful stimulators for the manifestation of ECM and integrin. Therefore they possess properties anticipated of wound cytokines and truly are among the most evaluated molecules in the wound-healing situation. VEGF-A was recognized as a key regulator of vasculogenesis and angiogenesis during development, denoting it was probably involved in controlling angiogenesis during wound-healing process [23]. Their enhancement in the wound area covered with Rhizochitin would be beneficial to wound-healing processes.

Furthermore, MMPs also display indispensible and beneficial roles in at a minimum of five foremost processes in natural wound healing [26]. It is sound proved that MMPs are necessary in the precise amount, in the correct place, and in the accurate time period (duration) for healing a wound. MMPs play crucial roles in removing damaged/devitalized ECM proteins, inducing angiogenesis, activating reepithelialization, contracting wound, and remodeling scar. However, clinical observation provides convincing proofs that unremittingly elevated levels of MMPs and other proteases preclude wounds from healing. Also, treatments diminishing MMP activities enhance wound healing that have hindered [27]. Therefore, it was suggested that decreasing the overexpression of MMPs in the wound area covered by Rhizochitin might lead to the protection of growth factors and acceleration of wound healing.

## Conclusion

A chitin-containing mycelial mattress of *Rhizopus stolonifer* obtained from a liquid static culture was utilized for wound dressing and biomedical use in this study. It was concluded that Rhizochitin so obtained was beneficial to wound healing. Possible mechanisms responsible for wound healing enhancement by Rhizochitin included decreasing the expression of PDGF in the proliferation stage, increasing the expression of TGF-β in the inflammation and proliferation stages, and increasing the expression of VEGF in the inflammation and proliferation stages. Rhizochitin also inhibited secretion of MMP-9 and MMP-2.
